# Fabrication of fluidic submicron-channels by pulsed laser-induced buckling of SiO_x_ films on fused silica

**DOI:** 10.1186/s11671-024-03987-w

**Published:** 2024-03-14

**Authors:** Nastaran Bakhtiari, Jürgen Ihlemann

**Affiliations:** Institut für Nanophotonik Göttingen e.V., Hans-Adolf-Krebs-Weg 1, 37077 Göttingen, Germany

**Keywords:** Silicon suboxide, Fused silica, Excimer laser, Micro/nanochannels, Micro/nanofluidics

## Abstract

Recently, considerable attention has been drawn to the field of micro/nanofluidic channels. However, current methods for fabricating micro/nanochannels are complex, costly, and time-intensive. In the present work, we successfully fabricated transparent submicron-channels on fused silica substrates (SiO_2_) using a straightforward laser process. To achieve this, a single-pulse excimer laser irradiation in a rear side configuration was employed to treat a thin film of UV-absorbing silicon suboxide (SiO_x_) through the transparent SiO_2_ substrate. A polydimethylsiloxane (PDMS) superstrate (coating layer) was applied over the SiO_x_ film before laser exposure, serving as a confinement for controlled structure formation induced by the laser. Under optimal laser fluence, the thin SiO_x_ film buckled, leading to the formation of channels with a width ranging from 10 to 20 µm and a height of 800 to 1200 nm, exhibiting a bell-like cross-sections following the so-called Euler buckling mode. Wider channels displayed morphologies resembling varicose or telephone cord modes. Subsequent high-temperature annealing led to the oxidation of SiO_x_, resulting transparent SiO_2_ channels on the fused silica substrate. The manufactured nanochannels exhibited promising potential for effectively transporting fluids of diverse viscosities. Various fluids were conveyed through these nanochannels via capillary action and in accordance with the Lucas-Washburn equation.

**Supplementary Information:**

The online version contains supplementary material available at 10.1186/s11671-024-03987-w.

## Introduction

Micro/nanofluidics is a field of science that deals with the control and transport of fluids restricted to structures on the micro/nanometer scale [1]. In recent decades, micro/nanofluidics has emerged in various areas, including biomedicine, lab-on-a-chip technology, heat transfer and chemical analysis [2–5]. As a result, numerous significant cases of interest to this multidisciplinary field have been studied at the micro/nanofluidic scale. Progress in micro/nanostructure manufacture strategies provides the capability to design a diversity of micro/nano fluidic geometries. Pores, pipettes and channels are three common fluidic platforms [6]. Compared to other structures, micro/nano channels play a more significant role due to their unique features such as substantial surface area-to-volume proportion, high adsorption and high permeability, etc. [7].

Micro/nanochannels are typically fabricated in planar structures using various methods. Depending on the manufacturing method, the dimensions of the micro/nanochannels can be controlled. So far several methods have been developed for manufacturing micro/nanochannels, including photolithography, wet chemical etching, electron beam lithography (EBL), and focused ion beam (FIB) milling [8–11]. However, most of these methods, particularly EBL and FIB, suffer from drawbacks such as the complexity and being expensive. Additionally, both EBL and FIB operate as serial writing processes, creating patterns point by point. This characteristic leads to comparatively slower fabrication speeds. While these methods offer high precision and resolution, the trade-off for this precision lies in the inherently slower processing speeds, a consequence of their serial nature. Hence, the interest in cost-effective, high-accuracy, and repeatable micro/nanofluidic channel fabrication techniques on a large scale is steadily increasing [12]. Unlike the aforementioned techniques, Laser direct writing (LDW) is a flexible and customizable method that enables the fabrication of micro/nano-scale structures over large substrate surfaces with high efficiency and low cost [6], moreover, direct laser writing can be used for serial or parallel processes. In the parallel method, in contrast to the serial approach, an entire pattern is exposed or processed simultaneously. This feature enables the creation of a complex fluid system, encompassing channels, tapers, splitters, and more, with just a single laser exposure—even with a single laser pulse.

In micro/nanofluidic systems, substrates like glass, silicon, and polymers are commonly used. However, due to opaque properties or exhibition of intrinsic fluorescence, silicon and polymers are often not suitable for the application in optical systems [6, 7]. In recent years, the manufacture of micro/nanochannels in fused-silica glass substrates were developed due to the unique properties of glass, including superior optical transparency, thermostability, biocompatibility, chemically inertness, mechanical strength, electrical insulation and hydrophilic property for chemical and biological studies and applications. However, fabricating micro/nanochannels on glass substrates is often done using expensive and complex processes that require high-class clean rooms [13, 14]. So, such hard and costly conditions have limited the development of glass micro/nanofluidic systems. Accordingly, alternative strategies for the manufacturing of micro/nanochannels on glass substrates are being investigated for the purpose of the development of micro/nanofluidics.

Micro/nanochannels employed in micro/nanofluidic applications may exist in either an open or closed configuration. Open micro/nanochannels have been created using various techniques [5, 9, 15], but the openness of these channels imposes certain constraints on their applications. Additionally, the fabrication of such channels frequently involves an etching process to eliminate debris generated during construction. Closed micro/nanochannels prove more advantageous for micro/nanofluidic applications, but the processes involved in their fabrication are frequently intricate and include multiple steps [16, 17]. One conventional approach for creating closed nanochannels involves glass-glass bonding, which can be achieved through methods such as anodic bonding or thermal bonding at high temperatures [18–20], as well as bonding methods at low temperatures [21–23]. While these techniques have proven effective in producing high-quality micro/nanochannels, they often face challenges due to their intricate procedural steps. These methods commonly entail precise surface refinement, etching, and complex bonding procedures.

High-temperature bonding methods, while offering robust strength, are generally more suited for fabricating micrometer-scale channels or nano-channels within thick substrates. On the other side, low-temperature bonding methods, despite not requiring elevated temperatures, exhibit drawbacks such as weaker bond strength and longer bonding process durations.

In the interim, bulging of thin film applied to a substrate through direct laser writing has presented an effective and bonding free method for the swift fabrication of closed micro/nanochannels: McDonald et al. [24] generated nano- and microfluidic channels by selectively delaminating thermally grown SiO_2_ films from Si100 substrates using a femtosecond pulsed laser. Janssens et al. synthesized nanocrystalline diamond (NCD) films within the reactor of a microwave plasma-assisted chemical vapor deposition (PACVD) system. Subsequently, these synthesized films were deposited onto a glass substrate. They then employed direct femtosecond laser writing to selectively etch fused silica, creating nanochannels within the substrate [25].

In this work, a direct laser writing method employing a single step parallel process has been developed for the fabrication of micro/nanofluidic channels on fused silica using a pulsed excimer laser. The process is related to the Laser-induced Forward Transfer (LIFT), which is a direct-writing method that requires exposing a donor material layer to laser irradiation. This exposure causes a laser-induced transformation, such as heating, melting, or ablation. As a consequence of this laser-induced effect, the donor material is transferred towards a positioned receiver substrate. This technique serves as a practical approach for producing micro/ nanostructures on surfaces [26–28], eliminating the necessity for clean room equipment. It is applicable to diverse classes of substrates, including glass. When fabricating micro/nanochannels on fused silica, two challenges arise:

First, laser ablation takes place exclusively when the target substance can efficiently absorb laser radiation. However, in the case of fused silica (SiO_2_), due to its transparent nature, the absorption of laser radiation is negligible [29]. To address this challenge, we employed a thin coating of silicon suboxide (SiO_x_, x ≈ 1), known for its strong absorption of UV laser light [30], on the fused silica substrate as the primary material. Due to the transparency of fused silica, rear-side irradiation (i.e. the irradiation of the coating through the substrate) can be used to induce film bulging. Moreover, our previous research has demonstrated that SiO_x_ can undergo oxidation to form SiO_2_, a fully transparent material, through a specific annealing process [31, 32]. As a result, transparent micro/nanochannels can be achieved on fused silica.

Secondly, undesirable deformations that occur during the manufacturing of micro/nanochannel structures represent a significant issue [13]. In order to achieve a smooth, uninterrupted and homogenous bulge along the channel, we implemented shape control through confinement. In our previous work [29], we have shown that the shaping of laser-induced structures is governed by the confinement layer. This involved applying a layer of polydimethylsiloxane (PDMS), which was affixed to the absorbent layer through slight mechanical pressure. The film is exposed to irradiation through the substrate at an intensity high enough to cause the film material to push forward and create a bulge. Since SiO_2_ is highly transparent to the wavelength of the incoming laser pulse, the laser pulse is anticipated to penetrate the fused silica with minimal alteration, directing its energy primarily into the SiO_x_ film.

Thus, the approach outlined in this study is similar to the LIFT method. However, unlike LIFT, the use of a PDMS confinement layer ensures that the film segment is not entirely detached from the substrate but remains connected to the irradiation edges, a crucial aspect for channel formation. This method provides the advantage of a one-step process for channel fabrication. Moreover, in contrast to the bulk femtosecond laser machining of channels, it produces very minimal debris within the channels, preventing any obstruction or clogging during the fabrication process. It also proves to be a more cost-effective option when compared to a DUV lithographic tool with a similar optical setup for mask projection, owing to the use of a simpler projection objective.

Also, the investigation of the transport of fluids within fabricated channels with water was conducted. Despite its seemingly simple structure and chemistry, water has remained a subject of constant interest. Its elevated dielectric constant and polarizability make it capable of dissolving ionic solids, and water can also establish hydrogen-bonded networks that exhibit unique properties seldom encountered in other liquids. As a result, in the majority of micro/nanofluidic studies, water and aqueous solutions have been the primary focus. Additionally, here other fluids with varying viscosities were investigated for comparative analysis.

## Experimental

### Sample preparation

Layers of SiO_x_ film, 533 nm thick with x ≈ 1, were coated onto 2 mm thick fused silica substrates through ion beam sputtering (IBS) (Laseroptik GmbH). These coatings are characterized by their hardness and strong adhesion. They also exhibit significant absorption in the ultraviolet spectral range, with high absorption coefficient of around 2.7 × 10^5^ cm^−1^ at a wavelength of 248 nm [33].

The PDMS-confinement film was produced by blending a mixture with a 10:1 ratio of silicone elastomer base and silicone elastomer curing agent (Sylgard 184) in a plastic box. Subsequently, this mixture was subjected to vacuum conditions for 4 days at room temperature. The film, obtained with a thickness of 1 mm, was then cut and owing to the adhesive properties of the PDMS layer, it was placed on the SiO_x_ film layer by applying slight mechanical pressure.

### Laser process and micro/nanochannel fabrication

**Fig.1:**
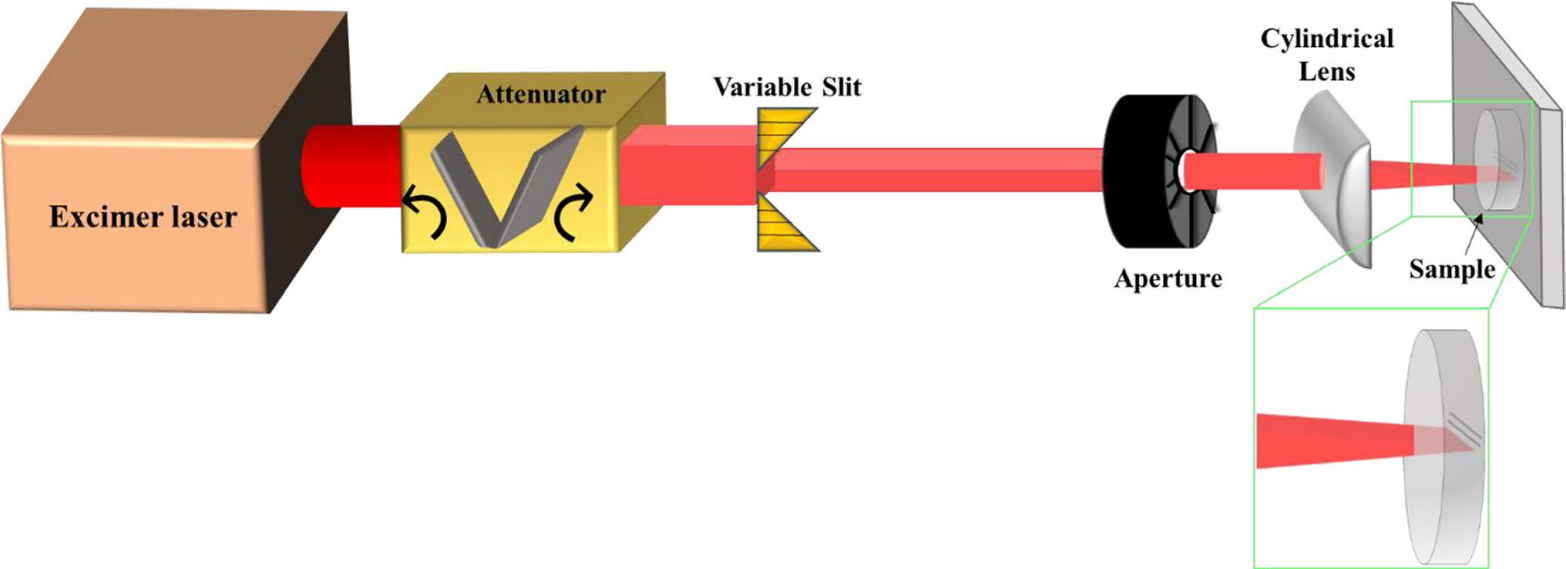
Schematic of optical setup employed to direct the laser onto the sample surface

The optical configuration employed to direct the laser beam onto the sample surface is illustrated in Fig. 1. The irradiation experiments were conducted utilizing a KrF excimer laser (Lambda Physik LPX 200) that emitted at 248 nm, featuring a pulse duration of 25 ns.

The laser light is directed through a variable slit and this light, is then projected onto the sample surface with 15× demagnification using a cylindrical lens with a focal length of 63 mm. In order to obtain optimal conditions for channels formation, the laser fluence was systematically changed using a variable attenuator. Additionally, the width of the channels was regulated from 10 to 50 µm by changing the slit size, and the channel length was kept at 5 mm by blocking a portion of the light with an aperture. Precise control over sample movement was achieved through the use of a three-axis motorized translation stage.

**Fig.2:**
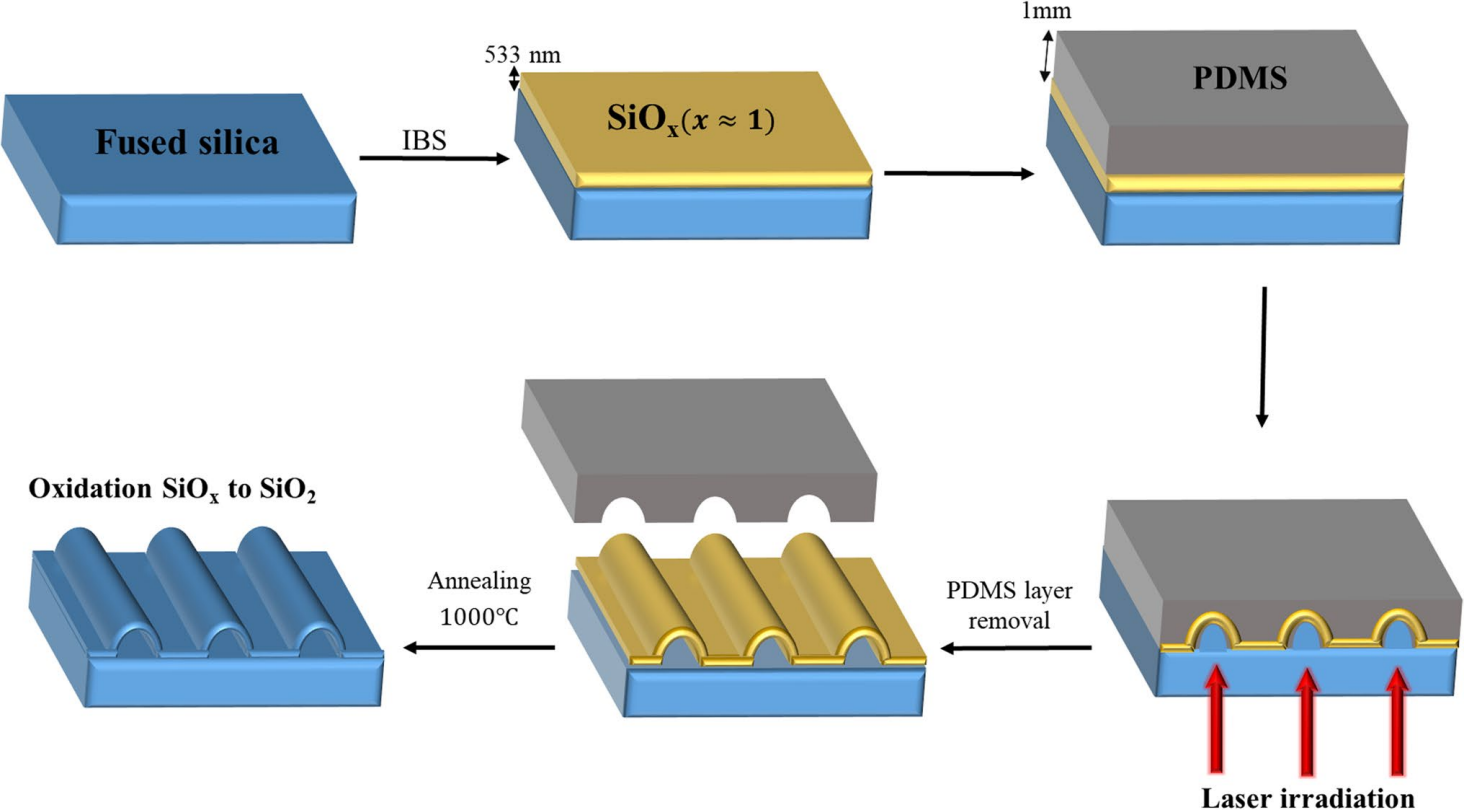
Schematic of the process steps used in the fabrication of channels

Figure 2 shows the steps of fabrication channels on the fused silica substrate. After the sample preparation and optical setup adjustments, the sample was exposed to a single-pulse radiation from the rear side. Subsequently, the PDMS layer was removed, and the sample was treated by thermal annealing at temperature of 1000 ℃ for 48 h in an air atmosphere.

### Characterization

The fabricated channels (before and after thermal annealing) were characterized by Optical Microscopy (OM, ZEISS Axio Imager.Z2m), Scanning Electron Microscopy (SEM, ZEISS EVO MA10) and surface stylus profilometer (Bruker, DektakXT). The measurement of contact angles for water, n-heptane and propylene carbonate (5 µL) on a SiO_2_ substrate was conducted using a contact angle analyzer apparatus (OCA 15 pro, DataPhysics) under ambient temperature conditions. Contact angles were measured at five different positions and the reported value represents the average.

### Fluidic transport

After the fabrication of channels, the sample was rotated by 90°, and a further laser exposure at a higher fluence, without the PDMS cover, was performed to ablate a film strip. This process was undertaken to prepare an opened cross-section for fluidic experiments and SEM analysis. Subsequently, the fluid-carrying capacity of the channels was determined by placing water, propylene carbonate, and n-heptane droplets near the edges of linear channels on the substrate. An optical microscope was then employed to record and analyze the fluid movements within these channels.

## Results and discussion

### Investigating optimal conditions in submicron-channel fabrication

**Fig. 3:**
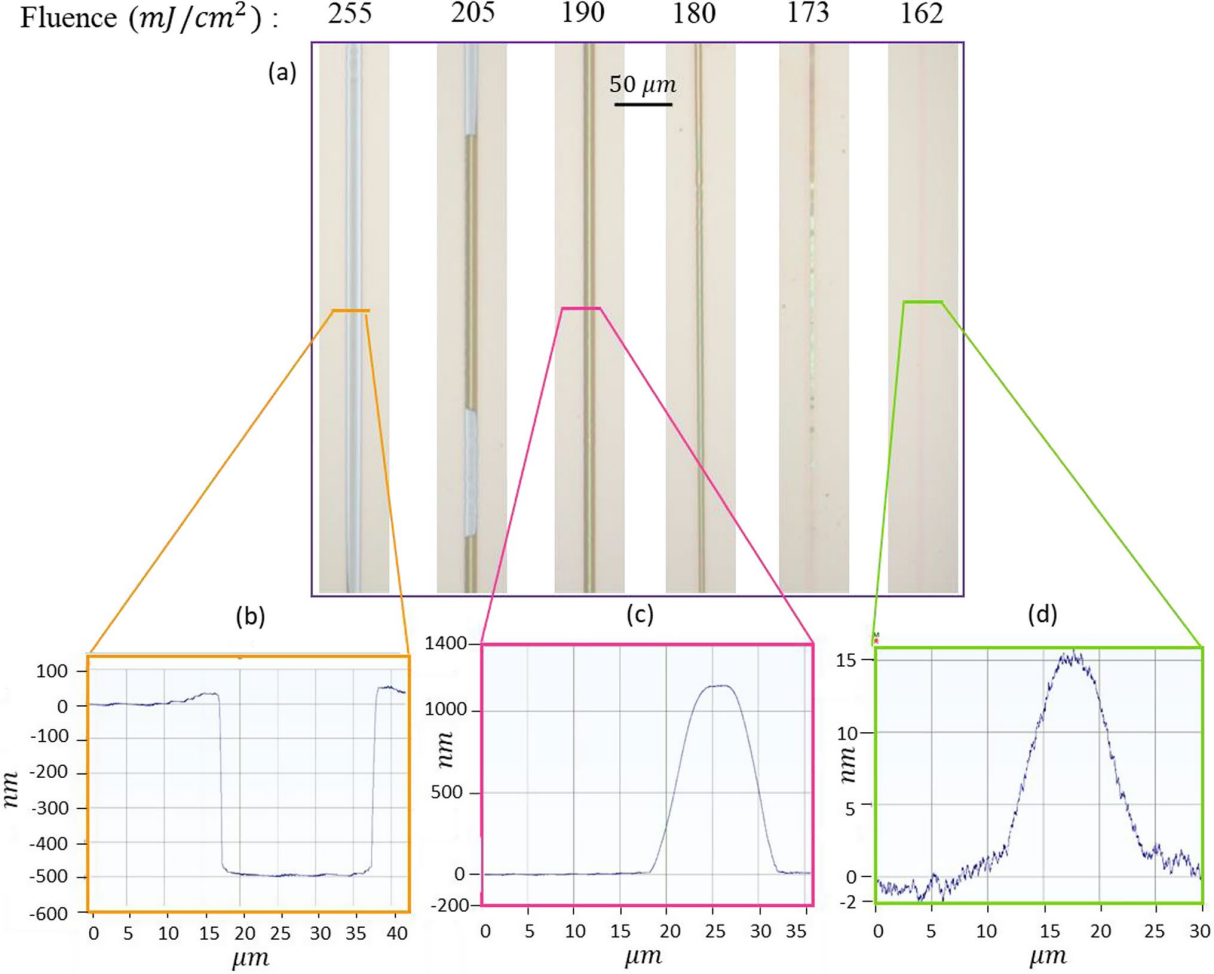
**a** OM images and **b**–**d** linear profiler analysis for the fluence range of 162 to 255 (${\text{mJ}}/{{\text{cm}}}^{2})$

The formation of channels is significantly influenced by the laser fluence. Laser fluence was determined by dividing the total energy input into the sample by the total illuminated field. Subsequently, it was systematically changed to obtain optimal conditions. Figure 3 illustrates the optical microscope images and linear profiler analysis for the range of fluence used. As previously mentioned, the SiO_x_ film is subjected to irradiation through the fused silica substrate. Under high fluence conditions, the film material becomes detached from the substrate and is propelled forward until film rupture takes place, leading to the removal of the film. The confirmation of this phenomenon is evidenced by the presence of a valley in the profile measurement, with a depth of approximately 500 nm, equivalent in terms of film thickness (Fig. 3b). In the optimal range of fluence (179–190 mJ/cm^2^), the film softens and stretches forward, giving rise to a homogeneous bulge in the presence of the PDMS confinement layer (Fig. 3c). At lower fluences, the film exhibits a slight and heterogeneous softening and bulge (Fig. 3d). Due to the transparency of SiO_2_ to the incident laser pulse’s wavelength, the pulse is anticipated to travel through the fused silica with minimal alteration, directing its energy entirely into the SiO_x_ film.

This laser energy absorption and subsequent SiO_x_ melting at the substrate–film interface initiate the initial delamination of the SiO_x_ film. The SiO_x_ film is conjectured to be heated and softened from the initial laser energy absorption and the heated film elevates throughout the entire irradiated area. Following the delamination, the film expands from the substrate, resulting from a combination of compressive stress relaxation within the film and transfer of momentum from the ablated material caused by the incident laser pulse [34]. When laser fluences surpass 205 ${\text{mJ}}/{{\text{cm}}}^{2}$, it is postulated that the force exerted on the film by the expanding ablated substrate material surpasses the film’s shear strength, leading to the complete detachment of the film from the substrate.

### Study on the dependency of channel height on laser fluence

**Fig. 4:**
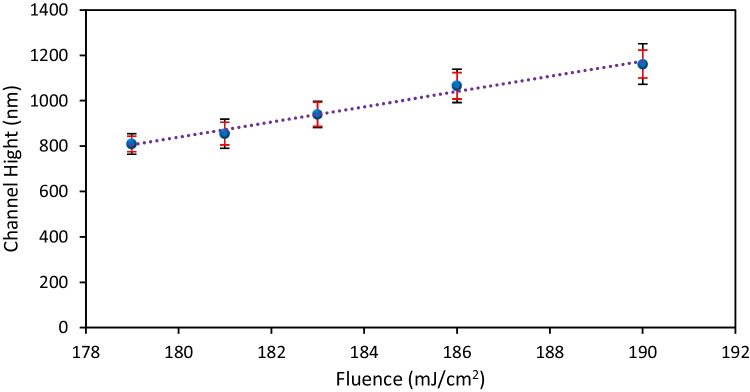
The measured channel height as a function of fluence. The black error bars illustrate the standard deviation in the height of fabricated channels, with a minimum of four channels produced under the same fluence. In contrast, the red error bars signify the standard deviation in height changes for each specific channel, measured at six points along the channel length

Further investigations revealed that while uniform channels are formed within the optimal fluence range, the channel height is a function of the laser fluence. Illustrated in Fig. 4, within the fluence range of 179 ${\text{mJ}}/{{\text{cm}}}^{2}$ to 190 ${\text{mJ}}/{{\text{cm}}}^{2}$, the channel height demonstrates a linear increase from 810 to 1160 nm. At higher laser fluences, the energy density is greater, leading to more intense interactions between the laser and the material. Elevated laser fluence can intensify material ablation due to the intense energy deposition. Conversely, increased fluence leads to higher temperatures in the irradiated region. This elevation in temperature might cause more localized heating and melting of the material. Consequently, this process pushes the film further, resulting in increased channel height. Therefore, varying the laser fluence offers a means to regulate the size of the channels. It is worth mentioning that a cylindrical lens has been used here because the limited image quality is sufficient. However, with the application of a high-quality imaging lens, it becomes possible to generate and optimize arbitrary profiles, for instance, by using gray scale masks.

### Effect of PDMS confinement layer

**Fig. 5:**
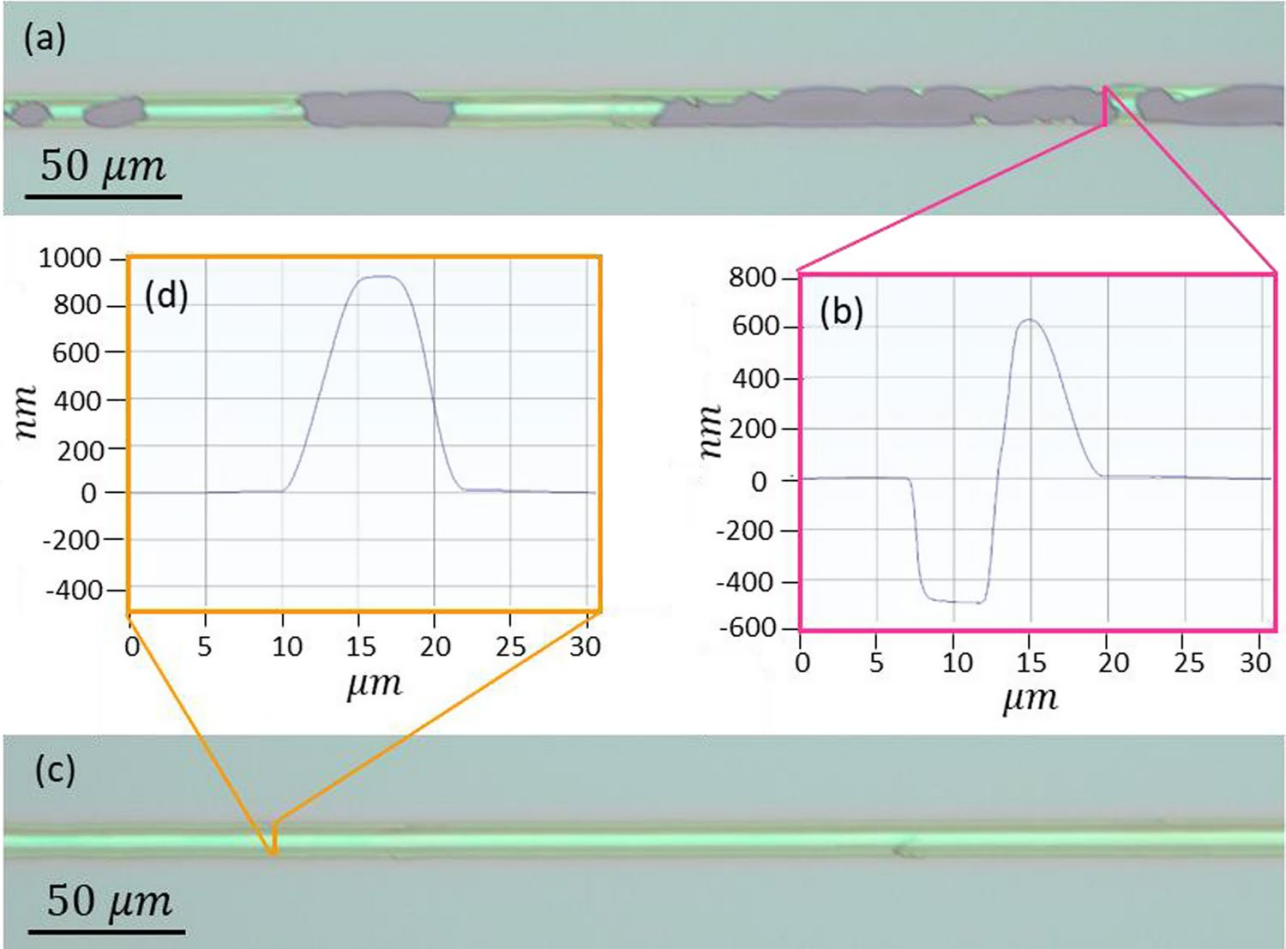
OM image **a** and linear profiler analysis **b** of channels fabricated without PDMS, and OM image **c** and linear profiler analysis **d** of channels fabricated with the PDMS confinement layer (followed by its removal) at a fluence of 181 ${\text{mJ}}/{{\text{cm}}}^{2}$

Figure 5 shows the OM images and linear profiler analysis of fabricated channels in the optimal fluence range, both with and without the PDMS confinement layer (subsequent to its removal). PDMS is a specific type of polymer based on silicon atoms, recognized for its optical transparency in the UV–VIS spectral region [35], although using the rear configuration for radiation does not require transparency and has enabled the use of a thick layer (1mm) with a certain degree of opacity. It is clearly evident that confinement leading to consistent and seamless features along the channels, as opposed to the irregular splatters noticed in the absence of confinement. In the previous section, it was highlighted that the radiating beam pulse moves through the SiO_2_ transparent layer with minimal attenuation and impacts the target material (SiO_x_). This interaction results in strong absorption and the rapid melting of the SiO_x_. The expansion of the materials exerts a driving force that tends to expel the molten substance. However, the neighboring confinement layer counteracts this by applying recoil pressure, restricting the free expansion of the softened material. This confinement effect leads to the formation of a coherent zone with a significantly reduced expansion velocity, effectively preventing the escape of material [36]. The utilization of a confinement layer thus results in a substantial improvement in the formation of channels. Simulation studies on the interaction of short-pulse lasers with materials have also demonstrated that spatial confinement provided by an overlayer prevents the explosive disintegration of the target [37].

Additionally, the outcomes of subsequent experiments demonstrated that the absence of the confinement PDMS layer prevents the creation of uniform channels, even when lower fluences are applied. Therefore, the utilization of the PDMS confinement layer seems to be essential for the uniformity of the channels.

### SEM characterization

**Fig. 6:**
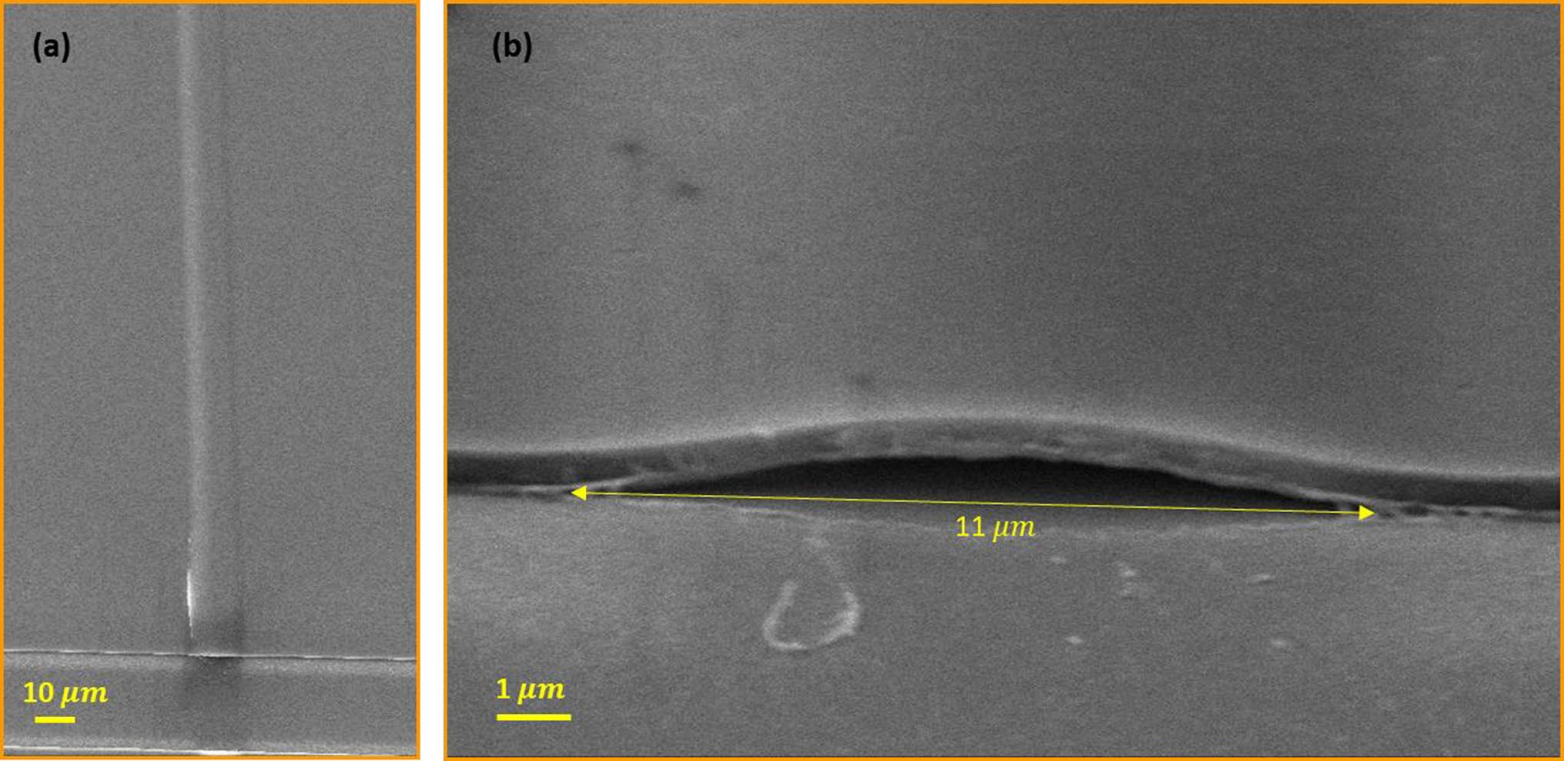
SEM images of the fabricated channel at a fluence of 181 ${\text{mJ}}/{{\text{cm}}}^{2}$ with a width of 11 µm and a height of 852 nm: **a** top view and **b** cross-section

To investigate channel formation between a SiO_x_ film and a SiO_2_ substrate using a single laser pulse, SEM cross-section and top-view images were conducted on a 11 μm width and 852 nm height channel after removing the PDMS layer (Fig. 6). The channel was created using a fluence of 181 ${\text{mJ}}/{{\text{cm}}}^{2}$. Figure 6a displays a uniform channel without any deformations. Additionally, the SEM cross-sections in Fig. 6b determined that the channel’s mouth is open highlighting the channel’s hollow nature.

### Effect of channel width on topography and morphology

**Fig. 7:**
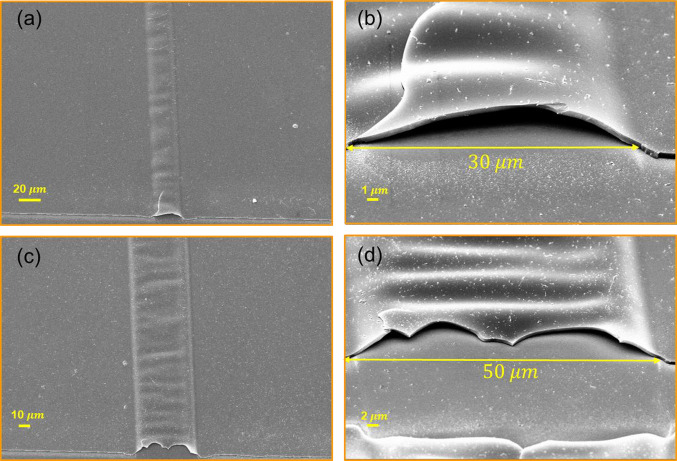
SEM images of the fabricated channel at a fluence of 181 ${\text{mJ}}/{{\text{cm}}}^{2}$ with a width of 30 μm: **a** top view and **b** cross-sectional; and 50 μm: **c** top view and **d** cross-sectional

A further investigation was carried out by altering the channel width by adjusting the slit size in the optical setup and examining its influence on the morphology and topography of the channels. Figure 7 displays SEM cross-sections and top-view images of channels measuring 30 and 50 µm. Figures 7a and c depict that despite the distortion of the upper surface of the channels, they remain closed throughout the path, while, the higher magnification images (Figs. 7b and d) reveal that the channel’s mouth with a 30 $\mathrm{\mu m}$ width is open. However, this aspect appears ambiguous for channels with a 50 µm width.

**Fig. 8:**
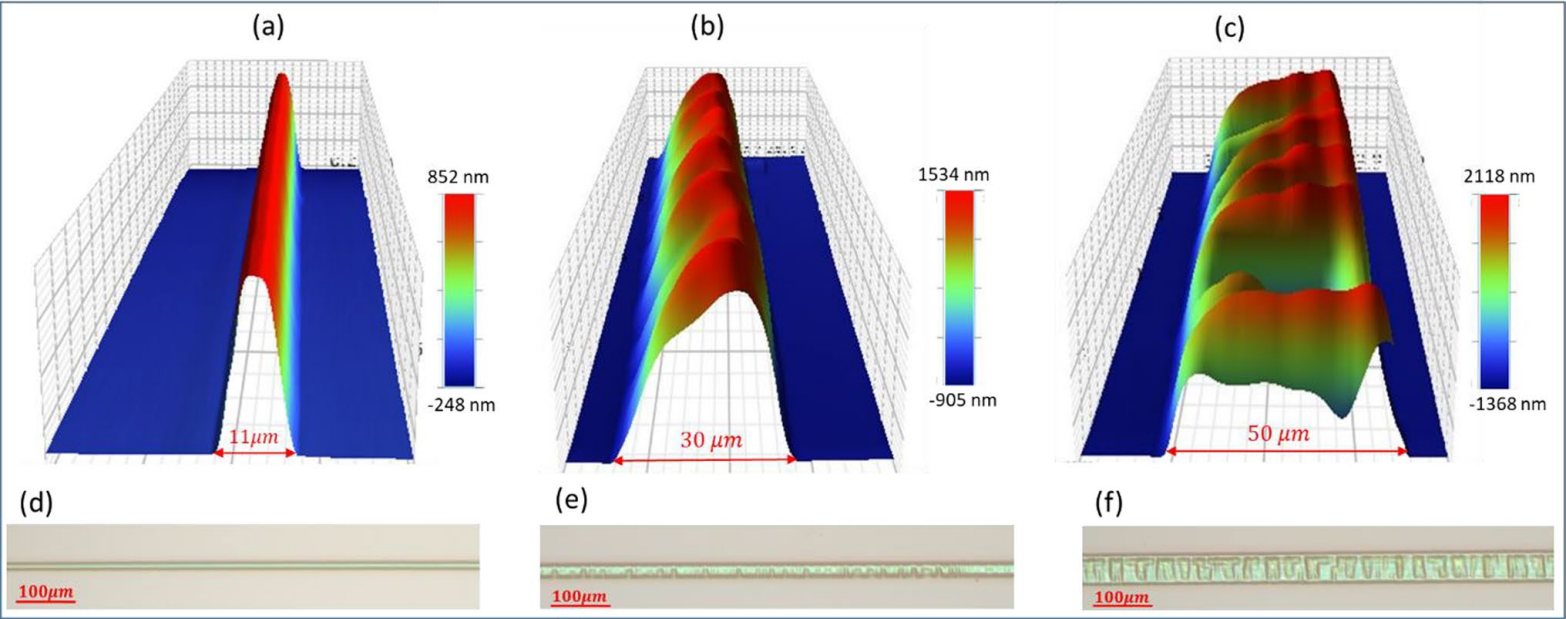
3D profiler images of the fabricated channel at a fluence of 181 ${\text{mJ}}/{{\text{cm}}}^{2}$: **a** 11 μm width (Euler mode), **b** 30 μm (varicose mode), **c** 50 μm (telephone cord mode). OM images of the fabricated channel: **d** 11 μm width (Euler mode), **e** 30 μm (varicose mode), **f** 50 μm (telephone cord mode)

The analysis conducted with the 3D profiler and optical microscope images provided additional insights into the distinct features of the channels produced at varying widths. As depicted in Fig. 8a, the channel with a 11 µm width showcases bell-like cross-section. In the classification of the buckle delamination of compressively stressed films, this is called the Euler mode [38]. The optical microscope image in Fig. 8d confirms the homogeneity of the channels over extended lengths. The results indicate that this structure remains consistent up to a width of approximately 20 µm. However, at higher widths, a change in the uniformity of channel shape becomes apparent. In such a way that, for channels with a width of 30 µm, the upper surface of the channel, i.e., the silicon suboxide film, changes its shape and shows a so-called “varicose mode” morphology (Fig. 8b, e) [38], and for channels with a width of 50 μm, it shows a “telephone cord mode” morphology (Fig. 8c, f). In the process of thin layer buckling on the substrate, the morphology of buckle delamination can vary based on the interface toughness, thickness, and stress of the film. Narrow strips tend to exhibit an Euler mode, medium strips show the varicose mode, while wider strips favor a morphology like to the telephone cord mode. The critical compressive biaxial stress of buckling $\left({\sigma }_{C}\right)$ is determined using the following equation [38, 39]:

1 $${\sigma }_{C}=\frac{{\pi }^{2}}{12} \frac{E}{{1-\nu }^{2}}{\left(\frac{h}{b}\right)}^{2}$$

where *E* and $\nu$ represent the Young’s modulus and Poisson’s ratio of the isotropic plate (film), *b* is half-width of the strip and *h* denotes its thickness. Young’s modulus defined as:

2 $$E=\frac{\sigma }{\varepsilon }$$

where *σ* is tensile stress and *ε* is tensile strain.

According to Eq. (1) when the strip width is narrow, the critical compressive biaxial stress of buckling increases, and Euler’s smooth mode primarily dominates the buckle morphology. Conversely, with wider strip widths, the critical compressive biaxial stress for buckling decreases, and the dominant morphology shifts to a wave-like varicose mode or telephone cord mode. On the other hand, narrower strip widths lack sufficient energy to overcome the film’s interface toughness, while wider widths provide the necessary energy to overcome this interface toughness. Since the other parameters of Eq. (1) are constant here, the critical stress changes with the change of the width of the channels, Hence, the expectation is for the formation of channels with bell-like cross-sections following the Euler mode within a specific range of widths (or stresses). As a result, the method used here provides channels ranging from 10 to 20 µm, featuring a smooth, uninterrupted, and homogeneous structure. However, the cross-sectional area (A) of the channel for the Euler mode is obtained from the following equation [38]:

3 $$A=bh\sqrt{\frac{4}{3}\left[{\left(\frac{b}{{b}_{0}}\right)}^{2}-1\right]}$$

here, ${b}_{0}$ represents the half-width associated with the onset of buckling, derived from Eq. (1) with ${\sigma }_{c}={\sigma }_{0},$ where ${\sigma }_{0}$ denotes the biaxial compressive stress in the unbuckled plate, referred to as the film stress. Consequently, the open amplitude of the channel between the film and the substrate can be adjusted by altering the film’s thickness in addition to changing the strip width.

### Thermal treatment

**Fig. 9:**
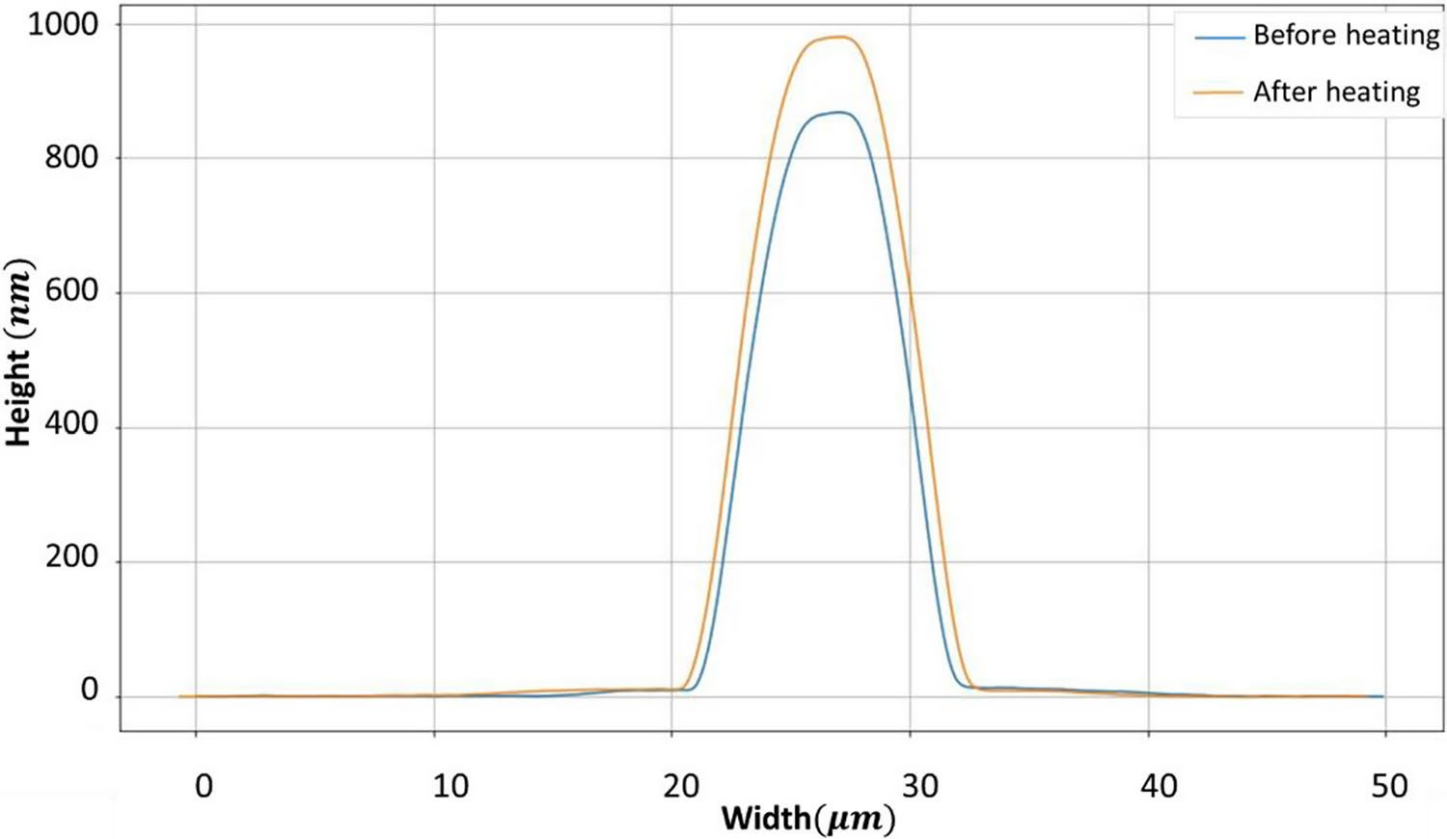
2D profiler of the fabricated channel before and after thermal annealing

After the removal of the PDMS layer and conducting the characterizations detailed in the previous section, the fabricated channels were subjected to thermal annealing at 1000 ℃ in air. This process aimed to oxidize the SiO_x_ layer, which is transparent in the visible but absorbs in the UV, to completely transparent SiO_2_. Figure 9 presents 3D profiler images of the fabricated submicron-channel with a width of 11 μm before and after thermal annealing. As it is clear, the channels maintained their bell-like cross-section during the heat treatment, with only the height increasing from 852 to 988 nm. It is well-established that thin layers of silicon suboxide undergo complete oxidation to silicon dioxide when subjected to thermal treatment in air and throughout the annealing process, the thickness of the silicon oxide layer experiences an increase [32]. During such annealing, the introduction of additional oxygen atoms results in an elevation of the stoichiometric index of the silicon oxide matrix, leading to change in atomic arrangement and bond structure. This change in stoichiometry enhances the volume of the layer, as silicon suboxide (SiO_x_) has nearly the same density as SiO_2_. When a SiO_x_ film undergoes oxidation to form SiO_2_, the ratio of the thickness of the resulting SiO_2_ film to the initial SiO_x_ film can be determined by the following equation [32, 40, 41]:

4 $$\frac{{h}_{{SiO}_{2}}}{{h}_{SiOx}}=\frac{{M}_{{SiO}_{2}}{\rho }_{SiOx}}{{M}_{SiOx}{\rho }_{{SiO}_{2}}}$$

where ${h}_{{SiO}_{2}}$ and ${h}_{SiOx}$ are thickness of the replaced SiO_2_ and SiO_x_ film. ${M}_{{SiO}_{2}}$ and ${M}_{SiOx}$ represent the molecular weights of SiO_2_ and SiO_x_ film and ${\rho }_{{SiO}_{2}}$ and ${\rho }_{SiOx}$ are the densities of SiO_2_ and SiO_x_, respectively. Assuming *x* is approximately 1, the calculated thickness ratio of silicon dioxide to silicon suboxide layer is approximately 1.35. This value aligns with the experimental result obtained here for the height difference of the channels. Therefore, the increase in the height of the channels after heat treatment is attributed to this phenomenon.

### Fluidic transport

**Fig. 10:**
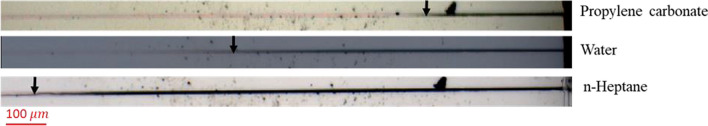
OM images of the transport of n-heptane, water, and propylene carbonate within 11 μm width channels after 500 ms. The black arrows illustrate the forefront of the fluid flow

As mentioned, under optimal conditions, there was a uniform deposition of energy over long lengths for channels. Consequently, the channel with a width of 11 μm and a height of 988 nm (after thermal annealing), fabricated under a fluence of 181 ${\text{mJ}}/{{\text{cm}}}^{2}$, was chosen for conducting fluidic experiments. To determine the fluid-carrying capacity of the channels, droplets of water, n-heptane, and propylene carbonate were positioned near the entrance of the channels. Observations through an optical microscope confirmed that the channels successfully filled with the specified fluids. Films obtained from the fluidic transfer process were then analyzed to determine the flow rate. As shown in Fig. 10 and videos S1 to S3 for n-heptane, water and propylene carbonate, the fabricated channels exhibit the ability to transport all three fluids without external force.

It is well known that in channels with a low volume-to-surface ratio, capillary action leads to the spontaneous filling of the channels with a wetting fluid. The kinetics of fluid capillary penetration in a horizontal capillary is described by the Lucas and Washburn (LW) equation [42]:

5 $${l}^{2}=Kt$$

6 $$K=\left(r\frac{\gamma }{\eta }\frac{\mathit{cos}\theta }{2}\right)$$

where $l$ denotes the position of the liquid front, *t* is time, *r* signifies the capillary radius, *θ* is the liquid/channel contact angle, γ and $\eta$ represent liquid surface tension and liquid viscosity, respectively.

So the rate of liquid penetrating a horizontal capillary can be expressed as:

7 $$\frac{dl}{dt}=\frac{r}{\eta }\frac{\gamma }{4l}{\text{cos}}\theta$$

The coefficient *K* in the LW equation has been modified for rectangular channels with height H, where the width is significantly greater than this height [43]:

8 $$K=\left(H\frac{\gamma }{\eta }\frac{\mathit{cos}\theta }{3}\right)$$

**Fig. 11:**
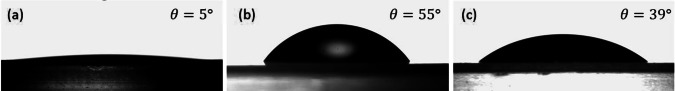
Contact angle of **a** n-heptane, **b** water and **c** propylene carbonate on the SiO_2_ surface

**Fig. 12:**
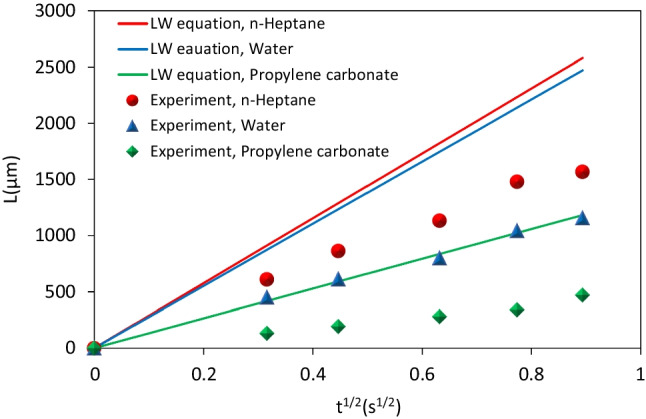
Comparing LW model predictions with experimental results in channels with a width of 11 μm and a center height of 988 nm during capillary filling

Since fabricated channel exhibits a profile where the height varies, being smaller near the edges and gradually increasing towards the center, to capture this geometry in capillary flow calculations an average height was used, which is half of the maximum height encountered at the channel center. This modification ensures a more adequate application of the Lucas-Washburn equation to fabricated channels. Figure 11 illustrates the contact angle observed for n-heptane, water, and propylene carbonate on the SiO_2_ surface. It is evident from the results that all three fluids exhibit wetting behavior on the sample surface. Consequently, it can be expected that the capillary penetration of fluid inside the channels is carried out spontaneously. The Table 1 provides the values of surface tension, contact angle, and viscosity for the examined fluids. As shown in Fig. 12 for all three fluids used, the rate of fluid penetration into the fabricated submicron-channels qualitatively follows the L-W equation and $l\propto \sqrt{t}$. Also consistent with the assigned value of *K*, the penetration rates for the fluids follow the order: n-heptane > water > propylene carbonate. Nevertheless, the actual penetration rates of fluids into the fabricated channels exhibit quantitative deviations from the L-W equation. In fact, the filling of micro/nanochannels by liquids occurs at a slower pace than what is predicted by the L-W equation. Various investigations have attributed the observed decrease in *K* to different physical phenomena, including variations in dynamic contact angle at the liquid/channel wall interface, electro-viscous effects, molecular structure and the emergence of a stagnant liquid layer near the walls [43–45].

**Table 1:** Physical properties of working fluids at 25 °C

Fluid	$\eta (mPa\times s)$	$\gamma (mN/m)$	$\theta^\circ$(this work)	Ref.
n-Heptane	0.39	19.9	5	[46]
Water	0.89	72	55	[47]
Propylene carbonate	2.53	34.6	39	[48]

Theoretically, clean glass is highly hydrophilic; however, various factors such as the absorption of atmospheric carbohydrate compounds, surface contamination, and chemical changes over time can lead to an increase in the water contact angle (WCA) on glass [49]. Consequently, the impact of the WCA on the kinetics of water movement in channels was investigated across a range of angles (20° to 60°) using the Lucas-Washburn equation. The results in Fig. S1 (in the supplementary material file) indicate that altering the contact angle within this range does not significantly affect fluid motion kinetics. Even at a WCA of 60°, liquid transfer remains facile within the channel. While enhancing hydrophilic properties can accelerate water movement within the channels, there is little cause for concern regarding the influence of increasing the substrate contact angle due to environmental factors on fluid movement.

## Conclusion and outlook

In the present study, we outline a technique for fabricating transparent channels with a scale of 1000 nm on fused silica substrates, specifically tailored for applications in micro/nanofluidics. To achieve this, a thin SiO_x_ film was applied to the fused silica substrate. Subsequently, the SiO_x_ layer was subjected to single-pulse excimer laser irradiation through the SiO_2_ substrate, along with the rear configuration and confinement with the PDMS layer. During laser irradiation, the SiO_x_ film undergoes heating and softening as a result of the initial absorption of laser energy. The heated film buckles across the entire irradiated area, leading to the formation of channels. To achieve completely transparent channels, after the removal of the PDMS layer, the sample underwent annealing at 1000 °C in an air atmosphere. This annealing process caused the oxidation of the SiO_x_ channels to SiO_2_ channels and their uniform integration with the substrate. Subsequent to the thermal treatment, the channels exhibited an increase in height, attributed to the introduction of additional oxygen atoms to the SiO_x_ during the heat treatment.

The height and width of the fabricated channels are functions of laser fluence and irradiation pattern. Channels with smaller widths displayed bell-like cross-sections, while wider channels exhibited varicose or telephone cord modes. The optimal fluence range for fabricating homogeneous channels with a bell-like cross-section was found to be between 179 and 190 ${\text{mJ}}/{{\text{cm}}}^{2}$, a parameter influenced by the thickness of the SiO_x_ film. Furthermore, an exploration of the potential of the fabricated micro/nanochannels for micro/nanofluidic applications revealed their capacity to facilitate the transfer of fluids characterized by diverse viscosities and surface tensions through capillary action, without the necessity for external forces. The qualitative behavior of fluid transfer inside the constructed channels follows the Lucas-Washburn equation.

In this work, a single submicron-channel has been successfully fabricated utilizing the capabilities of the excimer laser. While the immediate focus has been on this singular channel, it is crucial to emphasize the broader applicability of the employed methods. The functionality of the excimer laser, with its parallel processing capabilities similar to DUV lithography but more cost-effective, can seamlessly extend to the fabrication of complex patterns. Although the emphasis has been placed on a single channel in our current work, the methodologies and tools employed can be readily adapted to enable the production of intricate patterns through the incorporation of complex masks. This underscores the versatility and scalability of the techniques employed, allowing for a broader spectrum of applications beyond the restriction of a single channel.

Here, fluidic transfer has been accomplished through capillary action experiments. Nevertheless, the performance of these channels under high pressures remains uncertain, making it a potential subject for exploration in future research endeavors.

### Supplementary Information

Supplementary file. Fig. S1: Comparing the predicted values using the LW model for various WCAs with the experimental results

Supplementary file Videos S1–S3: Fluidics transfer processes include n-heptane, water, and propylene carbonate

Supplementary file. Video S2

Supplementary file. Video S3

## Data Availability

All data generated or analyzed during this study are included in this article and its supplementary information.
