# Supplementary material for: Fabrication of fluidic submicron-channels by pulsed laser-induced buckling of SiOx films on fused silica
Source: Discov Nano. 2024 Mar 14;19(1):46. doi: 10.1186/s11671-024-03987-w (PMC10940550; doi:10.1186/s11671-024-03987-w)
Supplement: Supplementary file 1 — Supplementary file. Fig. S1: Comparing the predicted values using the LW model for various WCAs with the experimental results [file 11671_2024_3987_MOESM1_ESM.docx]

**Fabrication of Fluidic Submicron-Channels by Pulsed Laser-Induced Buckling of SiO_x_ Films on Fused Silica**

Nastaran Bakhtiari *, Jürgen Ihlemann

Institut für Nanophotonik Göttingen e.V., Hans-Adolf-Krebs-Weg 1, 37077 Göttingen, Germany

*nastaran.bakhtiari@ifnano.de

**Fig. S1** Comparing the predicted values using the LW model for various water contact angles with the experimental results
